# Challenges Facing Radiation Oncologists in The Management of Older Cancer Patients: Consensus of The International Geriatric Radiotherapy Group

**DOI:** 10.3390/cancers11030371

**Published:** 2019-03-16

**Authors:** Tiberiu Popescu, Ulf Karlsson, Vincent Vinh-Hung, Lurdes Trigo, Juliette Thariat, Te Vuong, Brigitta G. Baumert, Micaela Motta, Alice Zamagni, Marta Bonet, Arthur Sun Myint, Pedro Carlos Lara, Nam P. Nguyen, Meritxell Arenas

**Affiliations:** 1 Department of Radiation Oncology, Prof. Dr. Ion Chricuta Oncology Institute, Cluj-Napoca 400015, Romania; tiberiu_popescu14@yahoo.com; 2 Department of Radiation Oncology, International Geriatric Radiotherapy Group, Washington, DC 20001, USA; karlssonulf30@gmail.com; 3 Department of Radiation Oncology, University Hospital of Martinique, Martinique 97200, France; anhxang@gmail.com; 4 Department of Radiation Oncology, Instituto Portugues de Oncologia Francisco Martins Porto E.P.E, Porto 4200-072, Portugal; lurdes.trigo@ipoporto.min-saude.pt; 5 Department of Radiation Oncology, Baclesse Cancer Center, Caen 14000, France; jthariat@gmail.com; 6 Department of Radiation Oncology, McGill University, Montreal H3T 1E2, Canada; te.vuong@mcgill.ca; 7 Department of Radiation Oncology, University of Bonn, Bonn 43217, Germany; brigitta.baumert@gmail.com; 8 Department of Radiation Oncology, Humanitas Gavazzeni Bergamo, Bergamo 24125, Italy; motta.micaela@libero.it; 9 Radiation Oncology Center, Department of Experimental, Diagnostic and Specialty Medicine, Sant’Orsola-Malpighi Hospital, University of Bologna, Bologna 40138, Italy; alice.zamagni4@unibo.it; 10 Department of Radiation Oncology, Arnau de Vilanova University Hospital, Lleida 25198, Spain; bonet.marta@gmail.com; 11 Department of Radiation Oncology, Clattterbridge Cancer Center, Liverpool CH63 4JY, UK; sun.myint@nhs.net; 12 Department of Radiation Oncology, Fernando Pessoa Canarias Las Palmas University, Las Palmas 35010, Spain; pedrocarlos.lara@ulpgc.es; 13 Department of Radiation Oncology, Howard University, Washington, DC 20060, USA; 14 Department of Radiation Oncology, Sant Joan de Reus University, University Rovira i Virgili, Tarragona 43204, Spain; meritxell.arenas@gmail.com

**Keywords:** older, discrimination, frailty, comorbidity, radiotherapy tolerance

## Abstract

The management of older cancer patients remains difficult because of data paucity. Radiation oncologists need to identify potential issues which could affect treatment of those patients. A workshop was organized in Barcelona among international radiation oncologists with special interest in the management of older cancer patients on April 22, 2018. The following consensus was reached: 1. Older cancer patients often faced unconscious discriminating bias from cancer specialists and institutions because of their chronological age. 2. Advances in radiotherapy techniques have allowed patients with multiple co-morbidities precluding surgery or systemic therapy to achieve potential cure in early disease stages. 3. The lack of biomarkers for frailty remains an impediment to future research. 4. Access to healthcare insurance and daily transportation remains an issue in many countries; 5. Hypofractionation, brachytherapy, or stereotactic techniques may be ideally suited for older cancer patients to minimize transportation issues and to improve tolerance to radiotherapy. 6. Patients with locally advanced disease who are mentally and physically fit should receive combined therapy for potential cure. 7. The role of systemic therapy alone or combined with radiotherapy for frail patients needs to be defined in future clinical trials because of targeted agents or immunotherapy may be less toxic compared to conventional chemotherapy.

## 1. Introduction

Management of older cancer patients remains a challenge as normal organ function declines over the years [1] and consequently, the number of co-morbidity factors increases with age [2]. Thus, invasive procedures such as curative surgery may be precluded in frail individuals with early stages disease [3]. On the other end of the spectrum, older cancer patients who are physically and mentally fit are often discriminated because of their chronological age and denied curative treatment because of the perception that they may not be able to tolerate treatment [4]. 

Older cancer patients referred for radiotherapy are often a mixed group of frail and physically fit patients who were initially evaluated by other physicians and thought to be candidates for palliative treatment only because of radiation toxicity [5]. On the other hand, some patients may have underlying frailty which may affect treatment outcome if undetected, thus highlighting the difficulty of their management because of the absence of clear guidelines. 

While historically it may be true, radiation oncology has evolved significantly over the past 20 years due to advances in imaging and radiotherapy techniques. This allows precise targeting of the tumor to a high dose and thus potential cure despite the presence of multiple co-morbidity factors [6]. As a result, radiation oncologists often face difficult treatment decisions on how to provide the best treatment to a growing segment of the population when they have to strike a delicate balance of choosing between treatment toxicity versus potential cure. 

Indeed, this issue was recently acknowledged by radiation oncology societies [7,8], some of which established their own older patients task force [9]. Still, modern radiotherapy treatment remains expensive because of the need for sophisticated linear accelerators which may not be available in emerging countries or limited access to health care insurance in developed ones [10]. 

A course of curative radiotherapy may last for six to eight weeks and may potentially affect an older patient’s quality of life (QOL) because of their limited mobility [11,12]. Those issues need to be addressed to provide potential solutions which may improve quality of care for older cancer patients, such as hypofractionated and stereotactic body radiation [6,13,14].

## 2. International Geriatric Radiotherapy Group Background

The International Geriatric Radiotherapy Group (IGRG) (www.igrg.org) was founded in 2012. At its inception, IGRG was meant to serve as a forum for radiation oncologists to discuss aging and its impact of aging on patient management. However, after multiple collaborative studies resulting in publications on the potential benefits of image-guided radiotherapy (IGRT) for normal organs sparing and improved local control for various types of cancer, the organization has morphed into a special interest and support group for older cancer patients [15,16,17,18]. Many workshops were organized during international cancer conferences. The first one was held in Rhodes in 2013 in affiliation with the International Institute of Anticancer Research (IIAR). A second workshop was conducted as an affiliate event at the American Society of Therapeutic Radiation Oncology (Astro) 2017 in San Diego. 

In the meantime, IGRG has grown from 15 institutions in three countries to 641 institutions in 103 countries, with over 700 members. The rapid growth and the request of new international members to become actively involved in prospective clinical trials through our open forums pushed the organization to develop a comprehensive policy towards promoting research not only for older cancer patients but also for minorities and women who are often excluded from clinical trials [19,20]. Thus, the third workshop focusing on the challenges facing radiation oncologists in the management of older cancer patients was organized on April 22, 2018 in Barcelona at the European Society of for Radiotherapy and Oncology (Estro).

## 3. Materials and Methods

Before the workshop, a thorough review of the literature through PubMed and Google Scholar search engines was performed to identify potential issues facing older cancer patients during radiotherapy. A panel of 14 international radiation oncologists discussed those issues through email and was able to identify six topics that needed to be addressed during the workshop:

1) Discrimination
2) Comorbidity
3) Tolerance to radiotherapy
4) Frailty
5) Access to medical insurance
6) Transportation.

Each topic generated a number of questions which would engage the audience into participating and proposing potential solutions. Prior to the workshop, the panel provided a presentation on the topic of discrimination of older cancer patients, in order to further educate the audience who may not be familiar with the literature.

Sixty persons attended the presentation, fifty participated in the workshop, and the others attended various meetings which were assigned to them by committee members at Estro. The workshop attendees were divided into six groups, which included two moderators, each discussing the topic that they selected. The moderators were members of the panel which designed the questions. At the end of the discussion, a representative of each group presented their conclusions and recommendations to the audience for further discussion, thus allowing the input of other members who may have a different opinion. A consensus was reached at the end of the workshop.

## 4. Consensus

Discrimination: Older cancer patients faced discrimination because of their chronological age. Unconscious physician bias and government healthcare policy hinder older cancer patients from obtaining early cancer diagnosis and optimal treatment, resulting in poor survival. 

Comorbidity and tolerance to radiotherapy: Despite the presence of multiple co-morbidity factors, older cancer patients tolerate radiotherapy very well with modern radiotherapy techniques and potential cure is attainable. 

Frailty: There is a paucity of biomarkers for frailty which impedes future clinical trials. Further research on biomarkers for frailty is needed to allow optimal treatment selection as the role of immunotherapy needs to be defined and investigated in future clinical trials for older cancer patients. Frailty may also hinder systemic therapy in patients with locally advanced disease requiring concurrent chemoradiation. But there are situations in which geriatric treatment would enhance the patient’s performance index and raise the chance for a curative treatment. Hence, the need for a geriatrician in the multidisciplinary team or for access to geriatric expertise

Access to medical insurance and transportation: The lack of access to medical insurance in many countries and socio-economic factors might prevent older cancer patients from seeking radiotherapy treatment. Those same issues may also affect daily transportation, which is further compounded by the limited mobility of the older patients and the presence of co-morbidity factors. However, hypofractionation, brachytherapy or stereotactic techniques may be ideally suited for older cancer patients to minimize transportation issue and treatment cost.

## 5. Recommendations

Current policy on cancer diagnosis and screening should take into consideration that the recommended upper age limit for screening should be revisited to allow early cancer diagnosis and optimal treatment. Physicians should be educated about the bias in discriminating older cancer patients. Hypofractionation may be ideally suited for older cancer patients to minimize transportation issue and treatment cost. Future research should be focused on finding new biomarkers for frailty.

The IGRG is planning to play a major role in promoting policy changes to improve cancer diagnosis, better access to medical insurance and transportation, education for health care providers in the management of older cancer patients, and to conduct prospective trials for optimal management of those patients.

## 6. Discussion

Older cancer patients often experience difficulty in accessing excellence of care. Surgeons and oncologists, including radiation oncologists, were reported to be reluctant to treat an older fit cancer patient aggressively because of their chronological age [21]. In addition, cancer in older patients is often diagnosed at a locally advanced or metastatic stage because current government policy frequently imposes an upper age limit for cancer screening [22,23]. This discrimination occurred despite the fact that over 60% of all cancers occurred in people older than 65, and by 2030, 20% of the U.S. population will be in that age group [24].

In addition, many clinical studies purposely excluded older cancer patients by design even though those patients may benefit from treatment despite their chronological age. For example, in a randomized trial for prostate cancer, patients who were 70 years-old or older were excluded from the study [25]. Furthermore, adding insult to injury, younger patients with a poorer performance status were enrolled, thus, highlighting physicians bias against older patients [26]. Stringent inclusion criteria, presence of multiple co-morbidities, frailty, and reluctance of physicians to enroll older patients into clinical trials were often cited as a barrier to their recruitment [27]. 

However, despite the presence of multiple co-morbidity factors and frailty which excludes other treatment modality such as surgery, older cancer patients tolerated radiotherapy very well, and in early stage disease, modern radiotherapy techniques such as stereotactic body radiotherapy (SBRT) and brachytherapy (BT), cure is feasible [6,28,29]. 

As an illustration, the combination of high tumoral dose and rapid dose fall off from the target allows increased local control and minimal complications in patients undergoing SBRT for early stage non-small cell lung cancer (NSCLC) [30]. 

Another important curative treatment is the use of BT specifically for prostate, breast, cervical, endometrial, vaginal, and vulvar cancers as an alternative treatment for surgery because it is minimally invasive and well-tolerated by older cancer patients. For instance, BT provided excellent local control and survival in patients with early stage prostate cancer while minimizing morbidity [31]. In selected patients with early stage breast cancer, the treatment course for accelerated partial breast irradiation (APBI) only lasted for five days instead of the traditional six weeks of external beam postoperative irradiation [32]. In addition, APBI has been proven to be a cost-effective treatment which provided equal local control and excellent cosmesis compared to other techniques of external beam irradiation [33,34]. For example, compared to three-dimensional conformal radiotherapy (3-DCRT) and intensity-modulated radiotherapy (IMRT), for each 1000 breast cancer patients treated, APBI provided a saving cost of 0.7 to 6 and 5 to 14.9 million dollars respectively [33]. These examples illustrate how omitting radiotherapy after surgery for older breast cancer patients is no longer justified because of the high cost of surgical salvage and patient anxiety following local recurrences.

Among older patients undergoing BT, with the possible exception of skin cancer, the success of this procedure for gynecological cancers has been consistent and longest since early 20th century. For instance, older patients with cervical cancer benefited from BT not only for cure but also for effective palliation of pain and bleeding [35]. Another illustration is the effectiveness of BT to prevent local relapse after hysterectomy for endometrial cancer which is one of the most common cancers among older women [36]. Loco-regional recurrences were significantly higher among patients who did not receive adjuvant external beam and BT following surgery. Even among older patients who could not have surgery because of multiple co-morbidities, radiotherapy alone combining pelvic irradiation and BT provided excellent local control and survival for early stage disease and effective palliation for locally advanced stage [36].

Thus, chronological age and physical status should not be a contra-indication for curative radiation. However, in patients with locally advanced or metastatic cancer requiring systemic therapy in addition to radiotherapy, frailty may become an impediment to the combined treatment. Treatment toxicity may outweigh treatment benefit in the last scenario. 

There are currently many tools to assess frailty. However, their complexity ranges from one item (Frail scale) to multiple items, such as the Comprehensive Geriatric Assessment [37]. Although efforts have been made, there is still no consensus on how to adopt a specific tool for clinical trials [38]. Nonetheless, with respect to day-to-day practice, access to geriatric expertise as part of multidisciplinary team is essential and should be encouraged [39]. As frailty reflects a decline in the body physiology with age, biomarkers would be complementary to the already established tools for frailty assessment such as CGA. Biomarkers could be used to screen for frailty in older cancer patients and to monitor the potential impact of treatment on this patient population. Sarcopenia, inflammatory, genetic, and immunologic markers have been proposed as potential biomarkers, but their effectiveness to define frailty remains unclear [40,41]. Thus, more clinical research needs to be done in the future to validate how biomarkers could be integrated in older patient management. 

Recently, a new biomarker, urinary 8-oxo-7, 8-dihydroguanosine (8-oxoGsn) has emerged as a promising biomarker to assess the patient physiologic age as it increases progressively with age [42]. If validated, 8-oxoGsn could be employed to assess older cancer patients functional status to determine whether systemic therapy could be integrated with radiotherapy for patients with locally advanced or systemic disease. 

Preliminary studies have reported a good tolerance in older cancer patients treated with chemotherapy and radiotherapy [43,44]. For instance, there were no difference in survival or grade 3-4 toxicity between older head and neck cancer patients (median age 74, range 70-90) and younger ones treated with concurrent chemoradiation [44]. However, the study was retrospective and needs to be validated on a large scale with the participation of multiple institutions. Another potential solution to improve local control and survival for older cancer patients who need systemic treatment concurrently or sequentially with radiotherapy is an agent with less toxicity compared to conventional chemotherapy. 

Targeted therapy or, more recently, immunotherapy, might provide a safe and effective treatment because of its favorable therapeutic ratio [45,46]. As an illustration, immunotherapy was well tolerated with minimal toxicity among older patients with urothelial and renal cell carcinomas while the response rate was similar to younger patients [47]. 

In other tumors such as melanoma, survival has been reported to be superior for older cancer patients compared to younger patients, with no difference in toxicity when they were treated with immunotherapy [48]. Thus, immunotherapy may be an ideal systemic agent in combination with radiotherapy for older cancer patients due to the radiation-induced abscopal effect [49]. However, given the paucity of data, prospective studies should be performed in the future to assess the efficacy of immunotherapy and radiotherapy in older cancer patients. 

Even though advances in radiotherapy techniques and new systemic agents are changing the management of older cancer patients, many hurdles remain for them to access quality of care. 

Impaired mobility with aging and the presence of co-morbidity factors may be a barrier for daily transportation to radiotherapy centers [11]. In an international review of older cancer patients, transportation difficulties has been cited as one of the major reasons for the patient to decline treatment [50]. Indeed, even though most older patients in the U.S. are covered by Medicare, current reimbursement policy does not cover transportation either in wheelchair vans or by stretcher to doctor’s office. However, Medicaid (a state program for socio-economic disadvantaged patients sponsored by the federal government) does cover those costs. It is clear that given the nation aging population, future changes in Medicare rules are needed to provide older cancer patients the resources for quality treatment [51].

A potential solution to minimize transportation issue is currently being proposed in certain countries such as decentralization of radiotherapy centers to minimize traveling distance [52]. Those pilot studies, if successful, might provide a template for other countries to adopt such policy. 

Transportation difficulty is further compounded in emerging countries by the paucity of cancer centers. Traveling time from the patient home to the cancer center may take more than seven hours, which can be a burden for both patients and their families as a conventional radiotherapy treatment may last seven to eight weeks [53]. In order to minimize the cost and the inconvenience associated with a protracted treatment, hypofractionation may be ideally suited for older cancer patients. 

Preliminary data suggests that hypofractionation is an effective radiotherapy schedule with acceptable toxicity for older cancer patients either as a single modality or combined with chemotherapy [54,55,56]. Preliminary studies suggested that hypofractionation may be effective in both curative and palliative settings for local control and improvement of patient quality of life in older cancer patients with various types of tumor [57,58,59]. Thus, future clinical trials for older cancer patients should focus on hypofractionation as a curative or palliative modality with or without systemic therapy. 

Clinical trials should explore patients’ expectations and acceptance of toxicities associated with these new radiation modalities. Finally, in this patient population, it will be most useful to establish selection criteria that will lead to both better oncological outcomes and improved quality of life.

It is clear that government policy needs to change in order to provide quality of care for older cancer patients. The encouraging news is the recent National Institute of Health (NIH) requirement that all clinical trials should include older cancer patients to qualify for federal funding. Could a new policy lead to more clinical trials specifically designed for older cancer patients such as inclusion of co-morbidity, frailty assessment, and biomarkers? With over 600 institutions in 103 countries, the IGRG provides an international network for collaborative research to conduct such studies in the future.

## 7. Conclusions

Modern radiotherapy techniques allow curative treatment for older cancer patients if diagnosed at an early stage. Government policy needs to change for early diagnosis of cancer, funding for research, and support for older cancer patients during radiotherapy. Even though management for older cancer patients remains a challenge for radiation oncologists, future clinical research designed specifically for older cancer patients may help clinicians making appropriate treatment decisions based on relevant data.
